# Health literacy and loneliness among physically inactive Danes aged 18–65: a cross-sectional study

**DOI:** 10.3389/fpubh.2024.1386591

**Published:** 2024-10-30

**Authors:** Delal Sarmanlu, Iben Rask Heuck, Helle Terkildsen Maindal, Michelle H. Lim, Knud Ryom

**Affiliations:** ^1^Department of Public Health, Aarhus University, Aarhus, Denmark; ^2^Health Promotion Research, Copenhagen University Hospital – Steno Diabetes Center Copenhagen, Herlev, Denmark; ^3^Iverson Health Innovation Research Institute, Swinburne University of Technology, Melbourne, VIC, Australia; ^4^Prevention Research Collaboration, School of Public Health, University of Sydney, Sydney, NSW, Australia

**Keywords:** health literacy, loneliness, physical inactivity, public health, health promotion

## Abstract

**Introduction:**

Both physical inactivity and loneliness are public health threats bringing huge costs to society and quality of life. The two health challenges often co-exist, suggesting physically inactive and lonely individuals to be a high-risk group. Health literacy as a concept is understood as a modifiable health determinant, and it has been proposed for promoting equity in future health promotion.

**Aim:**

The aim of this study was to examine the association between health literacy and loneliness among physically inactive adults.

**Methods:**

A representative sample of 6,196 Danish adults, aged 18–65 years, was invited to a screening on a set of health outcomes for physical inactivity, which was based on the International Physical Activity Questionnaire-Short Form. A total of 1,033 adults were classified as physically inactive and therefore received the full questionnaire screening on a set of different health outcomes including the Health Literacy Questionnaire (HLQ) and the Three-Item Loneliness Scale (T-ILS). Two statistical approaches were applied: (1) health literacy expressed as nine different continuous variables corresponding to the domains of HLQ using logistic regressions analyses to examine the association between health literacy and loneliness; (2) health literacy expressed as nine different binary variables showing proportions of low literacy among lonely versus non-lonely participants. Statistical analyses were performed using Stata/IC version 16.1.

**Results:**

Among a sample of 1,010 physically inactive adults, 23.7% felt lonely with a T-ILS score below ≥7. Regression analyses predicted a negative association between health literacy and loneliness in all HLQ domains, after adjusting for gender, age, education, and occupation. Adjusted ORs ranged from 0.21 (95% CI: 0.16; 0.27) to 0.69 (95% CI: 0.57; 0.83) in domains 1–5 and 0.50 (95% CI: 0.41; 0.61) to 0.70 (95% CI: 0.55; 0.89) in domains 6–9. A similar pattern was found in the analysis with health literacy as a binary variable as the proportions of low health literacy were the highest among persons with loneliness in all HLQ domains.

**Conclusion:**

Even after adjusting for sociodemographic factors, a negative association was predicted between health literacy and loneliness in physically inactive adults. This suggests that strategies for improving physical activity among inactive individuals might be more effective if they include a focus on enhancing health literacy and addressing loneliness.

## Introduction

Physical inactivity (PI) is an increasing public health threat globally (1). PI challenges individuals’ wellbeing and quality of life and has great economic costs to societies (2). Research suggests that determinants on various levels such as education, occupation, and social environment play an important part in physical activity patterns (3–7). Furthermore, PI also contributes to inequalities in health as low socioeconomic groups are more vulnerable to PI (8).

Collectively, research points out that age, sex, health status, self-efficacy, and motivation all are associated with physical activity (9). Bauman et al. (9) suggested that socio-ecological models are important for understanding PI because they include both social and physical environments as equally important contributors, alongside individual factors. A recent narrative review on PI in Denmark has gathered insights into its complexity, highlighting factors like loneliness and health literacy, among others, that are linked to PI (10).

In cross-sectional studies, loneliness has been shown to be related to lower physical inactivity levels (8, 11–14). Loneliness can be understood as a negative, distressing response to an individual’s perceived discrepancy between actual and desired social relationships (8, 11, 14); thus, loneliness is a subjective experience that does not necessarily correspond to the number of social interactions available (14). A systematic review by Pels and Kleinert (15) found that loneliness reduced the likelihood of physical activity. Thus, it is plausible that increased physical activity could reduce loneliness (15). A large part of the existing loneliness research has focused on loneliness in old age (16), although a more recent study indicates that young adults may be lonelier (17).

Weak social networks, poor social support, and low health literacy are all barriers to promoting health. Health literacy is considered a modifiable determinant of health and is regarded by the World Health Organization (WHO) as an essential and possible equality-creating element in future health-promoting efforts (18, 19). Health literacy is generally described as people’s motivation, knowledge, and competencies to access, appraise, understand, and apply health information to make sound judgments and informed decisions in everyday life concerning their health (19). Therefore, understanding how health literacy influences loneliness in physically inactive individuals is essential for promoting better health and wellbeing. Thus, this study aims to examine the association between health literacy and loneliness in a sample of physically inactive Danes aged 18–65 years, to investigate the hypothesis: “Health literacy is negatively associated with loneliness in this particular risk group.”

## Methods

### Study design and data collection

The cross-sectional study is based on questionnaire data among physically inactive Danes aged 18–65. The questionnaire survey, developed by the Department of Public Health at Aarhus University, included 18 general questions inspired by the “How are you feeling?” survey, a large-scale national survey conducted by the Danish Regions (20). The questionnaire was developed and subsequently pilot-tested (21). Data collection was conducted by YouGov, a global public opinion and data company, in the fall of 2019. YouGov provided the sample for the study based on their panel, which consists of 80,000 Danes. A representative geographical and socioeconomic subsample of the panel was selected for this study (see Characteristics section).

The data collection consisted of two steps: First, the sample of 6,196 individuals was screened for physical inactivity using the short version of the validated and WHO-recommended ‘International Physical Activity Questionnaire’ (IPAQ) (22). The short form version IPAQ – Short Form (IPAQ-SF) has been translated and validated in Danish samples. The IPAQ-SF consists of seven questions regarding physical activity in the past 7 or 14 days (23). A 7-day period was used in this questionnaire as suggested by the authors (24). Though self-reported physical activity instruments are considered less reliable (25, 26), alternative instrument tools for measuring physical abilities are based on a physical test (e.g., agility or fitness level). Such tests are resource-demanding as they demand more staff/research hours to collect as to a questionnaire-based self-report (27, 28). Thus, we opted to use self-reported data as screening in other ways was not possible. The respondents were contacted by e-mail, and informed consent was obtained. The final study sample consists of respondents classified as being physically inactive, as well as having full information about them regarding loneliness based on calculated scores. A total of 23 respondents were excluded from the analyses due to missing data regarding loneliness.

### Characteristics of the participants

**Figure 1:**
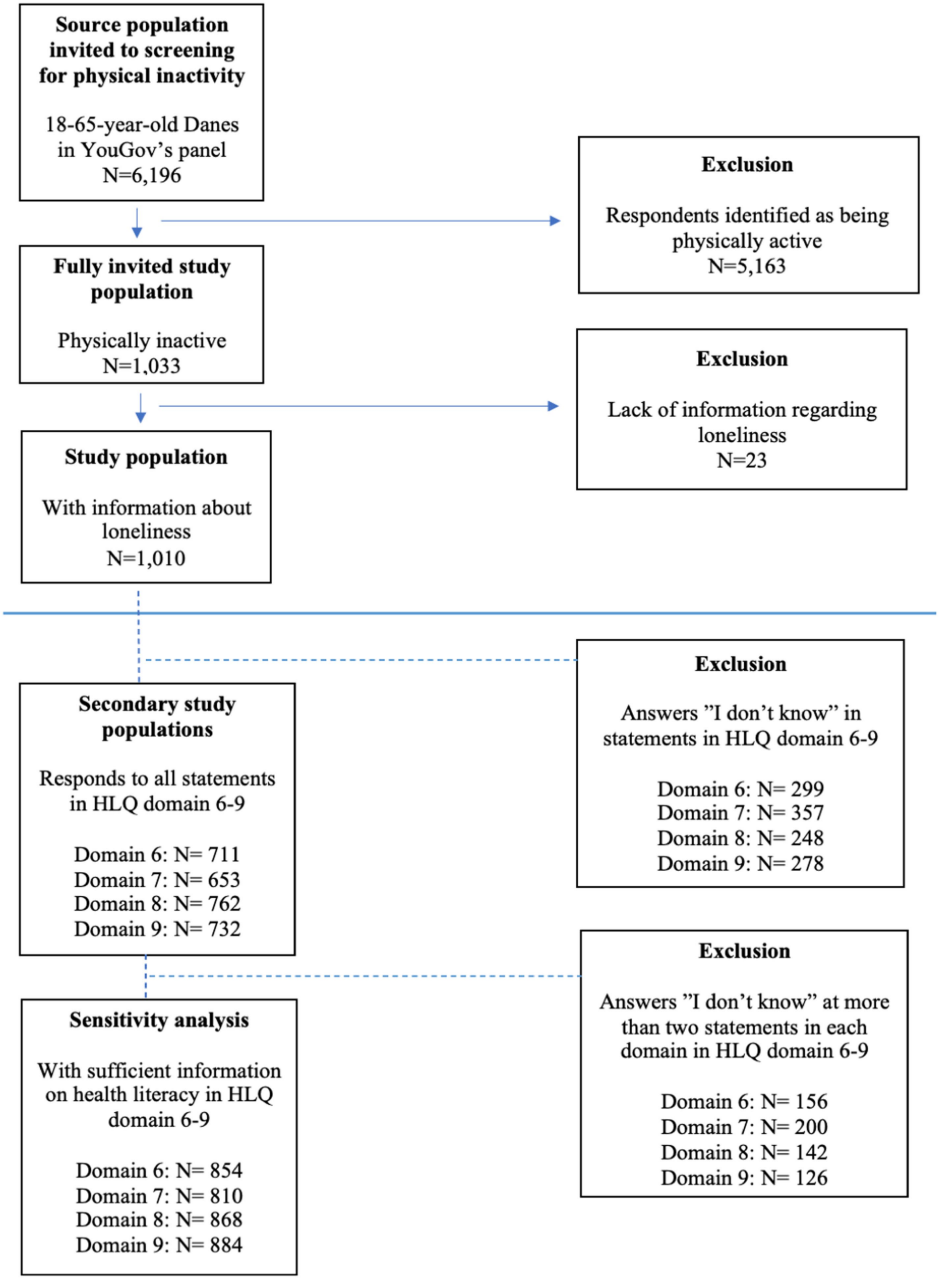
A flow-diagram of the study population.

The recruitment of the study sample was based on a representative source population from YouGov’s user panel. The source population consists of 6,196 Danes aged 18–65 corresponding to the working age (Figure 1), and it consists of more women (57.4%). An exception is the representation of ethnic minorities, which constitutes only 2–4% of YouGov’s user panel, compared to 14.4% in the Danish background population. A total of 1,033 individuals aged 18–65 were classified as being physically inactive based on the IPAQ-SF screening tool as mentioned above (see Figure 1). These individuals were included based on their answers indicating that they had not engaged in physical activity of either moderate or high intensity in the past 7 days. Thus, they were invited to answer the full questionnaire and were subsequently defined as physically inactive. These individuals formed the basis of the further study sample in this study.

### Measures

The Health Literacy Questionnaire (HLQ) was used to measure health literacy. HLQ is a validated questionnaire consisting of 44 items divided into nine domains representing different dimensions of health literacy (29). The HLQ has lately been introduced in large-scale surveys in many countries including Denmark. The Danish Health Authorities has added it to the large-scale “How are you feeling?” questionnaire, making it widely used. The HLQ contains 44 questions that cover 9 conceptually distinct areas of health literacy:

1. Feeling understood and supported by healthcare providers (four items).
2. Having sufficient information to manage my health (four items).
3. Actively managing my health (five items).
4. Social support for health (five items).
5. Appraisal of health information (five items).
6. Ability to actively engage with healthcare providers (five items).
7. Navigating the healthcare system (six items).
8. Ability to find good health information (five items).
9. Understand health information well enough to know what to do (five items).

For domains 1–5, the participants indicated their response using a Likert scale on the question: “How strongly do you agree or disagree with the following statements?” 1 = strongly agree, 2 = agree, 3 = disagree, and 4 = strongly disagree. On domains 6–9, participants indicated their response to the question: “How difficult or easy are the following tasks for you to do now?”; 1 = cannot do/always difficult, 2 = often difficult, 3 = sometimes difficult, 4 = often easy, and 5 = always easy. The tool has been translated and validated into Danish in 2016 (30). In this study, all nine domains were included. After YouGov’s instructions, an extra response category “do not know” was included. This led to exclusions and therefore a different number of observations in domains 6–9 than in domains 1–5.

The Three-Item Loneliness Scale (T-ILS) was used to measure loneliness. The T-ILS is a validated shortened version of one of the most widely used loneliness measurement tools, the UCLA Loneliness Scale (UCLA-LS) (31). The UCLA-LS has been translated and validated into the Danish context in 2006 (32). The T-ILS has shown a strong correlation with the full-scale tool and was developed specifically to measure loneliness in larger population surveys (33). Lately, T-ILS, like HLQ, has been included in the Danish Health Authorities “How are you feeling?” survey. The T-ILS consists of the following three questions: (1) “How often do you feel isolated from others?,” (2) “How often do you feel that you lack companionship?,” (3) “How often do you feel left out?.” The original response scale ranges from 1 to 3 on a Likert scale: 1 = never, 2 = sometimes, and 3 = always, although an extended response scale was used in this questionnaire: 1 = never, 2 = rarely, 3 = some of the time, 4 = often, 5 = always, and 6 = do not want to inform. This was done to align the T-ILS with the full questionnaire, facilitating easier reporting by the respondents.

To correspond with the original validated version of the questionnaire, a decision was made to combine the response categories “never” and “rarely” as well as “often” and “always,” so the total score ranges between 3 and 9. Loneliness was used as a dichotomous variable, as a score of ≥7.0 is defined as lonely, and < 7.1 is defined as non-lonely, consistent with other Danish population surveys (20, 34).

### Data analysis

In the description of the study sample, the distribution of the background variables of gender, age, level of education, employment status, marital status, and self-assessed health was displayed as stratified by loneliness in Table 1. The association between health literacy and loneliness among inactive adults was investigated in two ways. In the primary analysis, unadjusted and adjusted binary logistic regressions were used for each domain of health literacy. The variables for health literacy were included as continuous variables. In the secondary analysis, average scores for each domain, as well as proportions of low health literacy, were calculated. This was performed primarily to be able to compare the outcome with other studies. To calculate proportions, variables for health literacy were included as binary variables with cutoffs of ≤2 in domains 1–5 and ≤ 2.5 in domains 6–9, thus corresponding to previous studies (20, 35, 36). No adjustment was performed in the secondary analysis. Variables for health literacy expressed in domains 6–9 were based on a lower number of observations, because of expanding the scale with the response option of “I do not know.” In the primary analysis, a sensitivity analysis was performed including respondents with a maximum of two “I do not know” answers out of five to six statements, to examine the certainty of the estimates. The analyses were tested at a 5% significance level. All statistical analyses were performed using Stata/IC version 16.1.

**Table 1:** Participant sociodemographic characteristics and self-rated health stratified on loneliness (*N* = 1,010).

	Total	Non-lonely	Lonely	*p*-value
	*N* = 1,010 (100%)	*N* = 771 (76.3%)	*N* = 239 (23.7%)	
Gender *n*(%)				
Women	619(61.3)	458(59.4)	161(67.4)	0.027*
Men	391(38.7)	313(40.6)	78(32.6)	
Age *n*(%)				
18- to 29-year-old	128(12.7)	83(10.8)	45(18.8)	
30- to 39-year-old	190(18.8)	138(17.9)	52(21.8)	
40- to 49-year-old	245(24.3)	191(24.8)	54(22.6)	
50- to 59-year-old	280(27.7)	217(28.1)	63(26.4)	
60- to 65-year-old	167(16.5)	142(18.4)	25(10.5)	0.001*
Education level *n*(%)				
Primary school/do not wish to disclose	114(11.3)	78(10.1)	36(15.1)	
General or vocational high school education	139(13.8)	92(11.9)	47(19.7)	
Vocational education	239(23.7)	179(23.2)	60(25.1)	
Low-level education, under 3 years	124(12.3)	98(12.7)	26(10.9)	
Medium-level education, 3–4 years	261(25.8)	212(27.5)	49(20.5)	
High-level education, 5 years or more	133(13.2)	112(14.5)	21(8.8)	0.001*
Occupation *n*(%)				
Not working (retired/early retirement pay)	153(15.1)	104(13.5)	49(20.5)	
Not working	107(10.6)	59(7.7)	48(20.1)	
Student/apprentice/intern/trainee	85(8.4)	55(7,1)	30(12.6)	
Civil servant	420(41.6)	354(45.9)	66(27.6)	
Skilled/unskilled (non-civil servant)	168(16.6)	135(17.5)	33(13.8)	
Self-employed	33(3.3)	26(3.4)	7(2.9)	
Other	44(4.6)	38(4.9)	6(2.5)	<0.001*
Marital status *n*(%)				
Divorced	65(6.4)	43 (5.6)	22(9.2)	
Registered partnership/separated but still legally married or in a registered partnership	20(2.0)	13(1.7)	7(3.0)	
In a relationship but not cohabiting	76(7.5)	67(8.7)	9(3.8)	
Cohabiting but not married or in registered partnership	168(16.6)	127(16.5)	41(17.2)	
Married	400(39.6)	341(44.2)	59(24.7)	
Single	263(26.0)	169(21.9)	94(39.3)	
Widow/widower	18(1.8)	11(1.4)	7(2.9)	<0.001*
Self-rated health *n*(%)				
Poor	102(10.1)	52(6.7)	50(20.9)	
Less good	304(30.1)	200(25.9)	104(43.5)	
Well	501(49.6)	423(54.9)	78(32.6)	
Extremely well/excellent	103(10.2)	96(12.4)	7(2.9)	<0.001*

*p-value calculated by Pearson’s chi-square test with a 5% significance level.

## Findings

### Description of the study sample

The final study sample consisted of 1,010 individuals that provided information about loneliness. Their characteristics are displayed in Table 1. The majority were women (61.3%), aged 50- to 59-year-old (27.7%), had a medium-level education (25.8%), and were married (39.6%). Approximately half of the participants assessed their health as being good (49.6%), but only approximately 10% assessed their health as being very good or excellent.

A total of 23.7% of the study sample were classified as being lonely using the UCLA-LS-3 scores. Most of these individuals were women (67.4%). The proportion of 18- to 29-year-olds was 8 percentage points higher among lonely individuals, while the proportion of 60- to 65-year-olds was 7.9 percentage points lower among lonely individuals compared to non-lonely individuals. Lonely individuals have a lower level of education compared to non-lonely individuals. For instance, the proportion of individuals with high-level education was 14.5% among non-lonely individuals and only 8.8% among lonely individuals. Among lonely individuals, the proportion of non-employed on early retirement pay/retired and jobseekers were seven and six percentage points higher than among non-lonely individuals.

The proportion of functionaries was 18.3 percentage points higher among non-lonely individuals than among lonely individuals. Most non-lonely individuals were married, and most lonely individuals were single. Furthermore, more lonely individuals were divorced, and fewer were in a relationship. Finally, most non-lonely individuals assessed their health as being good (54.9%), while most lonely individuals assessed their health as being less good (43.5%). Among non-lonely individuals, 32.6% assessed their health as being less good or bad, while this applied to 64.4% among lonely individuals.

### The association between health literacy and loneliness

First, this section presents the primary analysis of the association between health literacy and loneliness by OR estimates (Table 2) as well as the sensitivity analysis of the domains 6–9 (Table 3). Subsequently, the secondary analysis of the average score and the proportions of low health literacy are presented (Table 4). Table 2 displays unadjusted and adjusted OR estimates for the association between the nine domains of health literacy and loneliness among physically inactive individuals.

**Table 2:** Unadjusted and adjusted logistic regressions of the association between the individual domains of health literacy and loneliness among physically inactive individuals (*N* = 1,010).

	Unadjusted			Adjusted*		
	OR	95% CI	*P*-value**	OR	95% CI	*P*-value**
Domain 1, *N* = 1,010	0.69	0.58;0.83	<0.001	0.69	0.57;0.83	<0.001
Domain 2, *N* = 1,010	0.55	0.42;0.71	<0.001	0.55	0.42;0.72	<0.001
Domain 3, *N* = 1,010	0.60	0.47;0.77	<0.001	0.58	0.45;0.75	<0.001
Domain 4, *N* = 1,010	0.25	0.20;0.32	<0.001	0.21	0.16;0.27	<0.001
Domain 5, *N* = 1,010	0.72	0.57;0.91	0.005	0.67	0.52;0.86	0.002
Domain 6, *N* = 711	0.47	0.39;0.57	<0.001	0.50	0.41;0.62	<0.001
Domain 7, *N* = 653	0.49	0.39;0.62	<0.001	0.51	0.40;0.65	<0.001
Domain 8, *N* = 762	0.70	0.56;0.88	0.003	0.70	0.55;0.89	0.004
Domain 9, *N* = 732	0.63	0.50;0.80	<0.001	0.66	0.51;0.85	0.001

*Adjusted for gender, age, level of education, and employment status.

**p-values are calculated by *z*-test for no effect with 5% significance level.

The HLQ covers nine conceptually distinct areas of health literacy: 1. Feeling understood and supported by healthcare providers (four items). 2. Having sufficient information to manage my health (four items). 3. Actively managing my health (five items). 4. Social support for health (five items). 5. Appraisal of health information (five items). 6. Ability to actively engage with healthcare providers (five items). 7. Navigating the healthcare system (six items). 8. Ability to find good health information (five items). 9. Understand health information well enough to know what to do (five items).

**Table 3:** Sensitivity analysis of OR estimates in domains 6–9.

	Unadjusted			Adjusted*		
	OR	95% CI	*P*-value**	OR	95% CI	*P*-value**
Domain 6, *N* = 854						
All “I do not know” coded as “Cannot/always difficult”	0.47	0.39;0.56	<0.001**	0.50	0.41;0.61	<0.001
All “I do not know” coded as “Always easy”	0.46	0.38;0.55	<0.001**	0.49	0.40;0.59	<0.001
Domain 7, *N* = 810						
All “I do not know” coded as “Cannot/always difficult”	0.48	0.39;0.60	<0.001**	0.53	0.43;0.66	<0.001
All “I do not know” coded as “Always easy”	0.48	0.39;0.59	<0.001**	0.51	0.41;0.64	<0.001
Domain 8, *N* = 868						
All “I do not know” coded as “Cannot/always difficult”	0.65	0.53;0.80	<0.001**	0.67	0.54;0.83	<0.001
All “I do not know” coded as “Always easy”	0.68	0.55;0.85	0.001**	0.70	0.56;0.88	0.002
Domain 9, *N* = 884						
All “I do not know” coded as “Cannot/always difficult”	0.61	0.50;0.75	<0.001**	0.64	0.51;0.80	<0.001
All “I do not know” coded as “Always easy”	0.65	0.53;0.81	<0.001**	0.68	0.54;0.86	0.001

*Adjusted for gender, age, level of education, and employment status.

**p-values are calculated by z-test for no effect with a 5% significance level.

Health literacy as continuous variables.

**Table 4:** Mean HLQ score and proportion with low health literacy (domains 1–5 ≤ 2, domains 6–9 ≤ 2.5) total and stratified on loneliness.

	Total, *N* = 1,010		Non-lonely, *N* = 771		Lonely, *N* = 239	
	Mean (SD)	% Low HL	Mean (SD)	% Low HL	Mean (SD)	% Low HL
Domain 1	2.53(0.81)	30.6	2.59(0.78)	28.1	2.35(0.87)	38.5
Domain 2	2.85(0.57)	11.3	2.91(0.55)	9.2	2.71(0.63)	18.0
Domain 3	2.38(0.61)	33.4	2.42(0.59)	30.5	2.24(0.65)	42.7
Domain 4	2.70(0.67)	17.7	2.84(0.60)	11.3	2.25(0.69)	38.5
Domain 5	2.47(0.63)	26.6	2.50(0.61)	24.9	2.37(0.68)	32.2

	Total, *N*=*		Non-lonely, *N*=**		Lonely, *N*=***	
	Mean (SD)	% Low HL	% Low HL	% Low HL	Mean (SD)	% Low HL
Domain 6	3.45(0.91)	15.9	3.60(0.85)	10.8	2.98(0.93)	32.0
Domain 7	3.30(0.83)	17.6	3.41(0.79)	13.7	2.93(0.83)	29.9
Domain 8	3.81(0.73)	5.6	3.85(0.72)	5.2	3.66(0.74)	6.9
Domain 9	3.75(0.71)	4.2	3.80(0.68)	3.5	3.57(0.74)	6.7

*domain 6 *n* = 711, domain 7 *n* = 653, domain 8 *n* = 762, and domain 9 *n* = 732.

**domain 6 *n* = 539, domain 7 *n* = 496, domain 8 *n* = 589, and domain 9 *n* = 567. *** domain 6 *n* = 172, domain 7 *n* = 157, domain 8 *n* = 173, and domain 9 *n* = 165.

The lowest difference in odds was predicted in domain 4 (social support) (OR = 0.21, 95% CI: 0.16; 0.27), while the largest relative difference in odds was predicted in domain 1 (feeling understood and supported by healthcare providers) (OR = 0.69, 95% CI: 0.57; 0.83) and domain 8 (ability to find good information) (OR = 0.70, 95% CI: 0.55; 0.89). This indicates that health literacy is the least negatively associated with loneliness in domains 1 and 8, and most negatively associated with loneliness in domain 4.

The sensitivity analysis displays that the OR estimates do not change significantly.

Table 4 exhibits an average score of health literacy as well as proportions with low health literacy for each domain stratified on loneliness. Low health literacy is defined as ≤2 in domains 1–5 and ≤ 2.5 in domains 6–9.

The results showed that lower mean scores for health literacy occur among lonely individuals compared to non-lonely individuals in all nine domains of HLQ. Similarly, a higher proportion with low health literacy was seen among lonely individuals in all nine domains of HLQ.

## Discussion

Among the physically inactive study sample, 23.7% were classified as reporting loneliness. A negative association between health literacy and loneliness in all health literacy domains was found, after adjusting for gender, age, education, and occupation. Thus, health literacy was found to be negatively associated with loneliness in this Danish population of physically inactive individuals.

The proportion of loneliness among physically inactive individuals found in this study (23.7%) was higher than in the Danish general background population measured in 2017 (8.6%) (28), which is in accordance with existing studies (37, 38). Age may be a significant contributing factor, as we found that the proportion of 18- to 29-year-olds was higher among lonely individuals, while the proportion of 60- to 65-year-olds is lower among lonely individuals than among non-lonely individuals. This indicates that young physically inactive adults are at risk of being lonely at the same time, which is also in line with another study conducted among Danes (34). Likewise, a previous study has also found a negative association between health literacy and loneliness (20), as found in this study among 18- to 65-year-olds. Furthermore, a recent study found that increased loneliness during the pandemic may have worsened physical health and health literacy outcomes among people in Australia (39).

In a Danish population-based study, 7% of the background population has low health literacy related to domain 6 (the ability to actively engage with healthcare providers). In contrast, the current study sample shows a prevalence of 15.9%, with as 32.0% among those who are simultaneously lonely (20). At domain 9 (understand health information well enough to know what to do), smaller differences with low health literacy are seen for 5% of the background population, while the corresponding numbers for the entire study sample are 4.2% for physically inactive individuals and 6.7% for the subgroup of lonely individuals. Though the large-scale population-based study (20) only measured domains 6 and 9, in contrast to all nine domains in this study, the difference between the background population and this study’s target group is still striking. Generally, this suggests that physically inactive and lonely individuals report lower health literacy that involves a social aspect (being able to interact with healthcare professionals).

The distribution of loneliness shows that the proportion with low health literacy is more distinct among lonely individuals at all levels. This trend is similar to the findings from a study by Manera et al. (40) which showed that associations between poor physical activity, sedentary behavior, and mortality were amplified by social isolation in a large UK study involving 497.544 participants. Though there has been an influx of new studies on loneliness due to COVID-19, only a few studies looked at health literacy and physical activity (39, 41). Thus, the results of this study are unique and provide insight into important mechanisms for physically inactive individuals. Consequently, there is a need to target physically inactive individuals in preventive public health strategies (40).

Studies investigating physical inactivity and loneliness have been significantly increasing during the COVID-19 pandemic, and these results point to an increase in both physical inactivity (42) and loneliness (43). Franke et al. emphasize that their findings on social attachment through physical activity are particularly relevant now due to the escalation of the incidence of social isolation and loneliness (44). O’Sullivan et al. further underscore the impact of the pandemic on loneliness attributable to major changes in individuals’ everyday lives and argue that governments and authorities should consider the wider social consequences of COVID-19, which may additionally be associated with poor health outcomes (45).

Building on these developments within physical inactivity and loneliness, our study provides additional insight into whether health literacy could be an important factor that influences the relationship between loneliness and physical inactivity. A hypothesis could be that loneliness is associated with low health literacy, which may lead to physical inactivity and ultimately poor health including premature mortality and morbidity. While these mechanisms cannot be tested in this study, it is possible to examine these associations between loneliness, health literacy, and physical inactivity in future research.

### Strengths and limitations

Since the study is cross-sectional, it is not possible to identify any causational associations since information on exposure and outcome has been obtained simultaneously. The results are therefore only applicable to predict whether there might be lower levels of health literacy among lonely adults who are at the same time physically inactive. The study population consists of a random sample of Danes aged 18–65 drawn from YouGov’s panel. A random sample is one of the best methods for obtaining a representative study population (46). The use of panel data ensured the representability and the size of the sample in this study, which is a strength. As mentioned, a limitation here is the lack of representation for ethnic minorities. Using panel data for data collection comes with pros and cons. It provides fast and easy access to participants, but there is a risk that participants might rush through the questionnaire to receive incentives. However, the panel data provider has software installed in their data collection system to ensure that respondents cannot choose answers randomly and complete the questionnaire inaccurately. Our analysis of the data has also ensured the quality of data. However, the panel data provided a dataset with no dropouts, though exclusions were made due to a lack of information, which occurred because of response categories allowing non-responses.

Additionally, self-reporting can be influenced by stigma related to the topics being asked about, which may affect the honesty of the answers. In this way, self-reporting itself can introduce measurement errors and misclassification. However, this risk is managed by using validated measurement tools to assess physical activity, health literacy, and loneliness. The questionnaire does not explicitly state that it measures, for example, health literacy, which means it is not explicitly stated for the respondent what is being measured. This approach may reduce some of the uncertainty stemming from differing knowledge bases and familiarity with the phenomenon. However, there is a risk that the excluded persons refuse to reply because of stigma or lack of competencies to answer the questions. This may have introduced selection bias since missing information on especially loneliness could be caused by stigma. It cannot be rejected that the missing information on health literacy is caused by stigma due to the wording of the statements, but it could also be caused by a lack of competencies to understand. This might have introduced selection bias.

In addition, it is a strength that all nine domains of HLQ are included in the questionnaire for measuring individual health literacy, providing a comprehensive and broad perspective on health literacy. Furthermore, it is a strength that the HLQ variables are used continuously as average scores for each domain in the logistic regression analyses, as recommended by the original authors. In the secondary analysis, a cutoff for low health literacy has been used. This has not been established and validated in the HLQ. However, several Danish studies have dichotomized the scoring of health literacy based on the domains 6 and 9 (20, 34). They have applied a cutoff of ≤2 on the response scale: 1 = very difficult, 2 = difficult, 3 = easy, and 4 = very easy. In Australia’s National Health Survey, a cutoff of ≤2.5 is applied on the response scale: 1 = cannot/always difficult, 2 = most often difficult, 3 = sometimes difficult, 4 = most often easy, and 5 = always easy (35). As the same response scale has been used in this study as in the Australian study, the cutoff of ≤2.5 has been applied to the domains 6–9. To follow the logic behind this, the cutoff for the domains 1–5 is set to ≤2 as such a score indicates predominant disagreement. It is important to point out that norms for answering questionnaires often are culturally determined, which is why validation of future cutoffs should also be validated to Danish norms.

A limitation of this study is selection bias according to gender as there are 619 women and 319 men included in this study. This type of bias could have an overrepresentation of women. Nevertheless, the data were adjusted for gender.

In analytical studies, the study population must be representative of the target population in relation to the context being investigated (46). The study population in this study comes from a random sample of Danes aged 18–65 drawn from YouGov’s panel. This is a strength as a random sample is one of the best methods for obtaining a representative study population (46). Furthermore, it is a strength that there are no dropouts, which is most likely due to the use of panel data, where the respondents received a reward for answering the questionnaire. Another strength is that the data on the HL measurement enabled a detailed analysis of the nine different continuous variables corresponding to the domains of HLQ. This made it possible to examine the association between HL and loneliness in a sample of 1,010 individuals.

A cutoff for low health literacy has not been established and validated in the HLQ. However, several Danish studies have dichotomized the scoring of health literacy based on domains 6 and 9 (20, 34). They have applied a cutoff of ≤2 on the response scale: 1 = very difficult, 2 = difficult, 3 = easy, and 4 = very easy. In Australia’s National Health Survey, a cutoff of ≤2.5 is applied on the response scale: 1 = cannot/always difficult, 2 = most often difficult, 3 = sometimes difficult, 4 = most often easy, and 5 = always easy, equivalent to the validated version (35). As the same response scale has been used in this study as in the Australian study, the cutoff of ≤2.5 has been applied to domains 6–9. To follow the logic behind this, the cutoff for the domains 1–5 is set to ≤2 as such a score indicates predominant disagreement. Yet, it is important to point out that norms for answering questionnaires are often culturally determined, which is why validation of future cutoffs should also be validated to Danish norms.

Furthermore, our findings are based on cross-sectional data, and therefore, no conclusions about temporality or causation can be made. In addition, it should be noted that there may be some imprecision and bias associated with using self-report measures of behavior in general.

Furthermore, another limitation of this study is the use of panel data. Using panel data for data collection comes with pros and cons. It gives fast and easy access, but there is a risk of participants just trying to finish as fast as possible to receive incentives. However, the panel data provider has software installed in their data collection system, ensuring that respondents cannot choose randomly and not filling in the questionnaire correctly. Our analysis of the data has also ensured us on the quality of data. Additionally, the use of panel data strengthened the representability and the size of the sample.

### Implications

The results of this study can support future research in the development and implementation of health-promoting interventions targeting physically inactive individuals. According to Nutbeam, higher health literacy leads to greater empowerment and autonomy, enabling individuals to take care of their own health and wellbeing (19). Efforts with a specific focus on promoting health literacy in physically inactive and lonely individuals will thus be ideal in terms of achieving behavioral change in the shape of more physical activity and social interaction. Furthermore, we believe that the results from this study hopefully could also be used to influence future directions in health education and the way health literacy is taught in schools.

Consistent with the “*Hvordan Har Du Det?*” survey (large-scale national Danish survey’s name in English: How Are You Feeling?), there may be an indication of intervening in relation to modifying and strengthening health literacy among physically inactive individuals in general and specifically among those individuals who are simultaneously challenged with loneliness. The results of this study further indicate that the intervention must be particularly targeted at strengthening the health literacy that deals with the ability to communicate with healthcare professionals as well as the use of social support and networks. According to Nutbeam (19), strengthening health literacy in relation to social support/networks is a prerequisite for critical health literacy. It can potentially be achieved through strengthening health literacy by learning in group-based efforts and through the involvement of family and significant others in networks. This could likewise promote distributed health literacy in the individual’s social environment. A similar format with a focus on physical activity has recently been recommended to alleviate loneliness (47), yet it has not been tested in combination with strengthening health literacy.

Our results suggest that most non-lonely individuals were also married, and most lonely individuals were single. In general, marital status is an important factor for both loneliness and physical inactivity (10). However, the UCLA-LS-3 does not assess different subtypes of loneliness such as intimate loneliness. Future research may consider administering the 9 items in the UCLA Loneliness Scale as it captures intimate connectedness (48) and has measurement invariance (46).

## Conclusion

From this study, we found that in a physically inactive population, higher health literacy is associated with a lower likelihood of being lonely. In addition, low health literacy is considerably prominent among physically inactive individuals who were also lonely, thus making health literacy an important area of focus for this high-risk group. The conclusion from this study also supports the subjective nature of loneliness among individuals and suggests health literacy as a possible key component to combat both physical inactivity and loneliness.

## Data Availability

The raw data supporting the conclusions of this article is not readily available because under Danish law, data cannot be shared without lawful agreement. Further inquiries can be directed to the corresponding author, upon reasonable request.
